# Factors influencing treatment default among patients initiated on antiretroviral at a district in the North West province of South Africa: A qualitative study

**DOI:** 10.4102/hsag.v31i0.3267

**Published:** 2026-05-14

**Authors:** Mmalebone R. Ntantiso, Maserapelo G. Serapelwane, Seepaneng S. Moloko-Phiri

**Affiliations:** 1 School of Nursing, Faculty of Health Sciences, North-West University, Mahikeng, South Africa

**Keywords:** antiretroviral treatment, human immunodeficiency virus, treatment default, patient, central chronic medicines dispensing and distribution, acquired immunodeficiency syndrome

## Abstract

**Background:**

People living with HIV who default on antiretroviral therapy pose a significant global and local health challenge, and South Africa is not immune to this challenge.

**Aim:**

This study explores factors influencing treatment default among patients receiving antiretroviral treatment in one district of North West province, South Africa. The study proffers recommendations to address default among patients initiated on anti-retroviral treatment.

**Setting:**

A clinical setting in the North West province, South Africa.

**Methods:**

A qualitative exploratory, descriptive and contextual research design was adopted. Purposive sampling was used to select potential participants. The population consisted of patients who defaulted on antiretroviral treatment and were registered at a specific primary healthcare facility. The sample size was determined by data saturation. Consequently, 12 participants who had defaulted on antiretroviral treatment for 3 months or more were interviewed. Data were collected through face to face, unstructured, in-depth individual interviews, field notes and an audio recorder. All ethical principles were followed throughout the study. Tesch’s method of analysis and open coding were employed to analyse the data.

**Results:**

Patients default on antiretroviral treatment for various reasons, including a deficient healthcare system marked by overcrowding and negative staff attitudes. Personal issues, unemployment and life partners emerged as reasons for default.

**Conclusion:**

Various factors influence the default on antiretroviral treatment, including access to healthcare facilities.

**Contribution:**

This study adds new knowledge about best nursing practices for managing patients who default on antiretroviral treatment.

## Background

Globally, there are 37.9 million people living with HIV, and 23.3 million of them are on anti-retroviral treatment (ART) (United Nations Programme on HIV and AIDS [UNAIDS] 2024:1). A study conducted by Berger et al. (2016:486) in the United States found that only half of the people diagnosed with HIV adhere to ART. This non-compliance places them at risk of deteriorating health and can often lead to death. Adherence to ART has been associated with improved viral suppression outcomes in HIV, while poor adherence to ART results in less effective viral suppression (Munyayi & Van Wyk 2021:2). The World Health Organization (WHO) recommends the 95-95-95 strategy (Nxumalo et al. 2025:1). This initiative aims for 95% of people living with HIV to know their status, 95% of those diagnosed to begin treatment and remain in care and 95% of people on treatment to achieve viral suppression by the year 2030. The Joint UNAIDS (2020:3) emphasises that reaching these targets would reduce the spread of HIV worldwide.

In India, an estimated 2.1 million people live with HIV, with half of them receiving ART at an adherence rate of about 81% (Hadaye, Jambhale & Shastri 2020:1922). Furthermore, the same study conducted in India at an adult ART clinic in a tertiary hospital confirmed that 5.6% of patients on ART missed treatment for a week, 5.3% missed treatment for 1–3 months, while 6.95% missed treatment for more than 3 months, and 10.9% skipped a full day dosage (Hadaye et al. 2020:1922).

In South Africa, the statistics show that 7.8 million people were diagnosed with HIV in 2024, and 6.3 million accessed ART (United States Agency for International Development [USAID] 2025). A confounding 4.3 million people have died from AIDS-related illnesses since the beginning of the epidemic. Although Benade et al. (2022:3) report that 59% of primary healthcare facilities in South Africa provide ART, treatment default remains a major concern. The South African Department of Health (DoH) plans to have an HIV-free generation by 2030 and states that ART should be administered to all infected people in the fight against the disease (2022:18). Despite the South African government’s effort to expand HIV treatment, defaulting on ART remains a critical challenge (Gilvydis et al. 2015:1275). However, some initiatives, such as fostering good relationships with health care providers and building a strong support system, including adherence clubs, have been established to increase synergy and retention. The Central Chronic Medicines Dispensing and Distribution (CCMDD) programme was introduced in 2014 to reduce long waiting times at primary healthcare facilities (Mabhena 2024). In this regard, CCMDD has demonstrated a positive influence on adherence to chronic medications, including ART (Benade et al. 2022:3). At our facility, the CCMDD service was already in place, and patients who met the eligibility criteria were enrolled in the programme.

In KwaZulu-Natal province in South Africa, a lack of trust in health care providers and insufficient psychological support are often the main barriers to retaining HIV and AIDS patients in care (Kave et al. 2019:14). In addition, a lack of trust results in disconnection that compromises patient communication with nurses and this results in male patients holding the perception that health care facilities are unfriendly to them and that they are mostly affirmative for women. Furthermore, a study conducted in Gauteng, South Africa, found that patients discontinue treatment because of transport costs to the facility, long queues and often-limited facility operating hours (Potsane 2023:1).

### Problem statement

In the selected district where the current study was conducted, a high proportion of patients miss their treatment appointments despite being initiated on ART (Tier.Net South Africa 2020).

According to the district clinical cascade, there is a challenge in meeting the district target to retain 77 235 patients on ART. Currently, there are 75 921 patients enrolled on ART, and only 89% are virally suppressed, leaving a gap of 1314 to reach the set target (Tier.Net South Africa 2020). Moreover, the district faces a significant challenge in ART enrolment and patient retention in care. Additionally, the authors of this study further observed that patients who have been on treatment longer tend to adhere more favourably than those who have only recently started treatment.

Furthermore, the statistics at the facility where the study was conducted show a high prevalence of HIV patients in the district (Tier.Net South Africa 2020). It is further reported that the facility has enrolled 4878 patients on ART, of whom 943 had already defaulted on treatment by December 2025 (Tier.Net South Africa 2020). This implies that the facility had more than 10% of patients who defaulted on antiretroviral treatment, exceeding its target of less than 5%. Additionally, there are limited studies on factors influencing the default rate among patients initiated on ART in the selected district of the North West province of South Africa. Hence, the purpose of this study is to explore and describe factors influencing treatment default among patients initiated on antiretroviral therapy in one district of North West province, South Africa. The results from this study could be used to reduce treatment default among patients diagnosed with HIV in primary healthcare facilities in the district.

## Research methods and design

### Design and methods

A qualitative, exploratory, descriptive and contextual research design was used to identify factors influencing treatment default among patients initiated on ART at a primary healthcare facility in the Dr Kenneth Kaunda district of North West province, South Africa. Purposive sampling was applied to select participants. In this regard, patients who defaulted on ART and agreed to be enrolled for the study were purposively selected to participate. The participants met the inclusion criteria if they were male or female with HIV aged 18 years and above, had defaulted ART for three or more consecutive months and could speak and understand local languages, which are English, Sesotho and Setswana. Data saturation was met after interviewing 12 participants.

### Recruitment of participants

The North-West University’s Health Research Ethics Committee (NWU-HREC) granted ethical approval under the reference number NWU-00216-22-A. Permission to conduct the study was granted by the North-West Provincial DoH. The gatekeeper, who was the operational manager, also provided a letter granting access to the health facility.

The operational manager appointed a mediator who was responsible for data capture and retrieval of patient data on Tier.Net (Klerksdorp, North West, South Africa). The mediator was requested to sign a confidentiality agreement. The researcher trained the mediator on the aims, methods and type of participants required and shared recruitment material. The mediator identified eligible prospective participants based on the inclusion and exclusion criteria. In this regard, a list of potential participants who had defaulted on ART at the facility was retrieved from the data capture on Tier.Net, and 225 ART defaulters were identified. All relevant files of participants who met the criteria were retrieved from the reception office to obtain recent contact details and home addresses, as most contacts were on voicemail.

The mediator recruited all potential participants telephonically, and the remainder was traced in person by the community health workers. The mediator referred those interested to an independent professional nurse and clinical preceptor with a Master’s degree in Nursing. The independent person met face to face with prospective participants at their respective residences on a specific date and time. The purpose of the appointment was to explain the purpose of the study and to sign consent forms to participate. Thereafter, the independent person provided the researcher with a list of the volunteers’ names and contact details, and the interview appointment dates were secured. All participants preferred to be interviewed at their own homes. The principle of autonomy, beneficence and justice was applied during recruitment and signing of the consent.

### Measures to ensure trustworthiness

Trustworthiness in research is about the collection of accurate data that are crucial in its fidelity to truth (Bryman & Bell 2015:717). In collecting data, the researcher ensured that findings are accurate by adhering to the principles of credibility, transferability, dependability and conformability. To enhance credibility, the researcher engaged with participants over a prolonged period, which involved participants’ paraphrasing their responses during interviews, asking probing and follow-up questions, seeking clarification and summarising statements to ensure accuracy. For transferability, the study setting and research methods used were fully described to enable other researchers and academic audiences to assesses the results. Dependability entails providing evidence that, if the study were conducted again with the same participants, the findings would be the same (Bryman & Bell 2015:717). The supervisors of this study verified the transcripts for consistency regarding the entire research process. Conformability refers to the potential for congruency of data in terms of accuracy, relevance or meaning (Connelly 2016:435). The study achieved conformability by involving a co-coder who performed an independent analysis of the data. The final validation of data was accomplished through a consensus meeting between the researcher and the co-coder.

### Data collection

Data were collected face to face by the researcher at the selected facility between May and July 2023. Unstructured in-depth individual interviews were used to collect data. A short demographic information questionnaire was completed by participants who provided informed consent. Data were collected from 12 participants. The two broad questions that guided the interviews are: (1) What factors influence treatment default among patients initiated on ART at a selected community health centre in Dr Kenneth Kaunda district of North West province, South Africa? (2) What recommendations could be made regarding treatment default among patients initiated on ART in a selected community health centre in Dr Kenneth Kaunda district of North West province, South Africa? The research questions were simplified in the interview guide.

Furthermore, to facilitate discussion, communication techniques, such as probing, clarification, paraphrasing and follow-up questions, were used. The study was piloted a week before the main study, and no modifications were made to the final instrument. The participants were requested to answer all questions freely without withholding any information. The researcher reminded each participant of their right to voluntary participation and that they could withdraw from the interview at any moment without penalty. The participants were also informed that the interview would last 35 min; however, the longest was 43 min, with a break in between to avoid fatigue. Before the interview, the researcher obtained consent from each participant to record the interview and addressed participants by identification letters rather than their real names. During the interview, no one experienced an emotional breakdown, and potential participants were reimbursed for their time and travel costs.

### Data analysis

Tesch’s eight-step approach (Creswell 2014:195) was followed by the researcher and the independent co-coder to analyse data. Firstly, the step was completed through verbatim transcription of all interviews. The researcher listened to all interviews on the tape recorder and transcribed the participants’ submissions word for word. Thereafter, all interviews and field notes were typed. Secondly, the researcher read all the transcripts carefully to familiarise themselves with the data. This helped the researcher to focus on what the participants said about the factors influencing treatment default by patients.

Tthirdly, the step included choosing one interview transcript that was shorter and more interesting and then reading it. The idea was to figure out its gist, search for transcripts with similar patterns and write these down. The task of reading and searching for similar information was applied to all interview documents. Fourthly, the step focused on reading all the transcripts and developing a list of themes. Similar themes were grouped and organised into columns, as main, unique and leftover themes. Fifthly, topics were abbreviated as codes, which were then written next to the corresponding text segments.

Sixthly, the researcher organised topics into alphabetical groups and condensed the list of categories by grouping-related topics. Seventhly, the description of themes addressing the study’s two research questions and objectives was presented in the qualitative narrative. This included an illustration of the table of themes and categories. The researcher met with the co-coder to confirm that the study’s results corresponded well with the collected data and to finalise the themes. Eighthly, the final step of analysis focused on interpreting the research findings and comparing them with existing literature.

### Ethical considerations

Ethical clearance to conduct this study was obtained from the North-West University’s Health Research Ethics Committee (NWU-HREC) (No. NWU-00216-22). The principle of beneficence was maintained by protecting all participants from physical, legal, emotional, social and financial harm (DoH 2015:14).

This was achieved by avoiding the asking of sensitive questions to participants. In this regard, the researcher focused on the study’s objectives and follow-up questions during data collection. Furthermore, the venue of the interview was secure and safe, with no unauthorised individuals allowed entry. If any participants suffered emotional distress during the data collection process, the researcher would have immediately ended the interview and referred the participant to a counsellor for support. However, no referrals were made during interviews, and all participants successfully completed their interviews.

The principle of justice states that all role players in research, including participants, should have a fair balance of risks and benefits (DoH 2015:14). To uphold this principle, participants who defaulted on ART were selected fairly and for reasons that were directly related to the research. No participant was treated unfairly. This principle of respect for persons was achieved by the following: (1) Before data collection, participants were presented with the aim and objectives of the study and signed an informed consent form as evidence of their voluntary and informed participation in this study and (2) to avoid intimidation, participants were informed of their right to participate as well as their right to withdraw or refuse participation without consequences.

## Results

All participants were Black, aged between 18 and 56 years. The sample comprised five males and seven females. Seven of the participants were employed, while five were unemployed. Ten participants were single, and two were married. The employment roles included petrol attendants (*n* = 3), domestic workers (*n* = 2) and contractors (*n* = 2). Educational attainment ranged from Grade 6 to N1.

Participants reported missing their treatment for periods ranging from 4 to 32 months. Living arrangements varied, with two participants residing with their parents and seven renting rooms in different households. All participants were ineligible for registration in the CCMDD programme because they were not virally suppressed, meaning their viral load had not been below 50 copies in the past 6 months. Additionally, they did not adhere to ART in the past 6 months.

Participants identified various factors influencing treatment default among patients initiated on ART in one district in the North West province of South Africa. Table 1 depicts themes, categories and sub-categories from the interviews conducted. Two main themes emerged: (1) factors influencing ART default and (2) suggestions for improving ART adherence. Firstly, entitled ‘factors influencing ART default’, four categories emerged with eight sub-categories. Secondly, participants offered suggestions to improve ART adherence. One category and three subcategories emerged from the second theme. Themes and sub-themes are outlined in Table 1, complemented by participant quotations.

**TABLE 1:** Results of the study.

Themes	Categories	Sub-categories
1.1 Factors influencing ART default	1.1.1 Personal factors	1.1.1.1 Experienced side effects of ART treatment 1.1.1.2 Financial challenges 1.1.1.3 Unsafe environment
	1.1.2 Employment factors	1.1.2.1 Patients’ work-related schedule
	1.1.3 Life partner-related factors	1.1.3.1 Lack of support from life partner
	1.1.4 Factors related to the poor health system	1.1.4.1 Overcrowding of patients and long waiting time 1.1.4.2 Nurses’ negative attitudes 1.1.4.3 Difficulty collecting ART treatment during the week 1.1.4.4 Poor infrastructure leading to a lack of privacy
2.1 Suggestions related to the improvement of adherence to ART treatment	2.1.1 Improvement of the health system	2.1.1.1 Treatment delivered to patients’ residential addresses 2.1.1.2 Access to ART on weekends 2.1.1.3 Nurses to demonstrate a good attitude 2.1.1.4 Improvement of infrastructure and implementing the fast lane model

ART, anti-retroviral treatment.

### Theme 1.1: Factors influencing anti-retroviral treatment default

Throughout, participants highlighted a variety of factors influencing treatment default among patients initiated on ART in the district. A discussion of categories that emerged from the first theme, which are personal factors, employment factors, life partner-related factors and factors related to the poor health system, is presented.

#### Category 1.1.1: Personal factors

The first category addresses personal factors that influenced patients to default on ART and consists of four subcategories, namely: participants experienced side effects of ART, financial challenges and unsafe environment. The following is a discussion of these subcategories.

**Sub-category 1.1.1.1: Experienced side effects of anti-retroviral treatment:** During discussions, participants highlighted persistent treatment-related side effects as a major factor influencing treatment default. The participant had severe vomiting, dizziness and prolonged inability to eat after taking the medication. Despite repeatedly raising these concerns, the participant indicated that she was advised to continue with treatment, suggesting a perceived lack of management of side effects by healthcare providers. In this regard, the participant had this to say:

‘Another reason is that since 2018, I raised a concern that I experience side effects when taking treatment. I was told to drink them as normal; however, there was no difference because I can’t get used to them, and so does my body. Maybe I’m thinking too much into this or something but when I drink them today the following day I can’t eat the whole day. When I drink them, I vomit and can’t even eat. I become dizzy, my granny told me that my system is dirty, then I did detox. I continued experiencing side effects even after detoxing. I last took treatment last year [*2022*] when my grandmother collected 2 months’ supply for me, I don’t remember the month.’ (Participant A, female, 25 years)

**Sub-category 1.1.1.2: Financial challenges:** The findings of this study indicate that financial challenges are a critical determinant of treatment default, particularly among unemployed participants. Limited income constrained participants’ ability to afford transport to healthcare facilities, directly impeding regular clinic attendance and resulting in extended periods without treatment.

This was reported by one of the participants who said:

‘Secondly it is because I lack money for transport. I live far away from the clinic and when it’s time to collect the treatment, I won’t be having any money. It’s because I don’t have money as I said that I only receive grant.’ (Participant D, female, 27 years)

The other participant further said:

‘It’s not nice mama to wait for long because sometimes you don’t even have two rands [*R2*] to buy an apple when you are hungry; I get to the consulting room without having energy even when the nurse says something I can’t hear anything because of hunger.’ (Participant C, female, 40 years)

**Sub-category 1.1.1.3: Unsafe environment:** The findings reflect that living in unsafe environments has a negative impact on adherence to ART. In this regard, participants reported exposure to risks such as mugging, gun violence, stabbing and rape in the areas where they resided, which led to them not returning to the clinic for treatment collection.

The participant had this to say:

‘Where I stay it’s a bit far from the clinic. I don’t mind walking back home, but the problem is that when I return home sometimes it’s dark and its dangerous. This place is very dangerous; we are afraid of one another on the road at night even during the day it’s not safe. When they mug you, it’s either they take your phone or stab you, sometimes people are raped.’ (Participant F, male, 35 years)

Another participant put it this way:

‘It’s not safe at night, there are gunshots everywhere especially when there is load shedding. Thieves and guns are everywhere here, even when you leave the house around 5am you are unsafe, we are always scared. In our section its worse, during sunset there are gunshots all the way.’ (Participant G, male, 56 years)

#### Category 1.1.2: Employment factors

The second category was employment factors, in which only one sub-category emerged: patients’ work-related schedules. The participant’s submission is presented here.

**Sub-category 1.1.2.1: Patients’ work-related schedule:** The findings of this study indicate that workplace-related constraints are a major contributor to treatment default among many participants. Inability to leave work for ART collection, largely because of inflexible work shifts and limited employer support, emerged as a problem. One of the participants said:

‘Firstly, it is because of the work-related issues. I once came and reported to clinic staff that my shifts were abnormal because I work at a filling station. I tried to get my mother to collect treatment on my behalf, but she fell ill and was the only one who could collect on my behalf.’ (Participant A, female, 25 years)

Another one put it this way:

‘I stopped collecting treatment because I struggled to get a job for a long time. When I got it as a new employee, I could not understand how things were done. My shifts were complex, I knocked off late hence I could not come for treatment collection.’ (Participant J, male, 37 years)

#### Category 1.1.3: Life partner-related factors

From this category, only one theme emerged, which is reported as follows:

**Sub-category 1.1.3.1: Lack of support from life partner:** In this study, despite male partners being aware of their own HIV-positive status, they prevented their loved ones from returning to the clinic for treatment. Hence, defaulting on ART was highly associated with a lack of support from life partners.

One of the participants said:

‘I used to collect my treatment accordingly. Then I met this other guy who told me that he doesn’t want a girlfriend who takes treatment. That’s when I stopped taking my treatment, so I stopped taking it because I love him.’ (Participant E, female, 28 years)

The same phenomenon was reported differently by another participant who said:

‘I don’t know what to say because whenever I plan to go to the clinic, he shouts at me. He says I am selfish and only take care of myself, yet he hasn’t even started taking his treatment and he doesn’t like taking pills. He said such a word to me. I’m afraid of my partner, he will see the date on my appointment card that I’m taking treatment. Iyooo … it is going to cause trouble.’ (Participant B, female, 18 years)

#### Category 1.1.4: Factors related to the poor health system

How ART services were rendered and coordinated affected patients’ adherence. This includes overcrowding, long patient wait times and nurses’ negative attitudes. Study participants discussed the individual factors that influenced their decision to default on ART; these subcategories are discussed independently.

**Sub-category 1.1.4.1: Overcrowding of patients and long waiting time:** Participants reported poor adherence to ART follow-up visits because of long clinic queues, resulting in extended waiting periods. It was perceived that the nursing staff deliberately delayed services, resulting in imposed and prolonged waiting times.

The quotations that support overcrowding and long waiting times are as follows:

‘There isn’t much I can say because the reason we don’t come to the clinic is due to the long waiting times. I feel like they are doing it deliberately to keep us long because there is nothing we can do. Okay, I leave home at 6:30am because I stay nearby but I end up leaving around 16:00pm or 18:00pm just like the person who arrived at 12:00pm and by that time there’s no progress.’ (Participant I, female, 23 years)

Another participant put it this way:

‘Itjooooo! We wake up very early, sometimes you wake up at 5am and you queue until 12H00, at 3pm you are still at the clinic to collect medication, yet you are not sick.k.’ (Participant C, female, 40 years)

**Sub-category 1.1.4.2: Nurses’ negative attitudes:** Participants described being shouted at, judged or reprimanded without an opportunity to explain the circumstances that led to ART default. Such interactions were perceived as emotionally harmful and stigmatising reinforcing feelings of shame and fear rather than encouraging re-engagement with care.

One of the participants had this to say:

‘By the time I arrive at the consulting room, because I defaulted, the nurses ask me questions in a hurtful manner as to why I am not taking my treatment and I won’t have any answer because of the way they ask me. I feel hurt because instead of them encouraging me or talking to me in a good manner they say hurtful words.’ (Participant D, female, 27 years)

Another one put it this way:

‘According to my view, nurses like shouting without getting the full story of what went wrong. They just think for themselves, they don’t give us a chance to explain what happened. They just shout, they don’t ask why you are doing this or why the situation is looking like this.’ (Participant B, female, 18 years)

**Sub-category 1.1.4.3: Difficulty in collecting anti-retroviral treatment during the week:** The participants highlighted the lack of availability of ART services during weekends, which restricts timely access to treatment. This suggests that routine services, such as ART collection, are confined to weekdays, with weekend care reserved only for emergency or severe cases.

One of the participants had this to say:

‘We can’t be assisted on weekends, the only people who will get help are the ones that need serious hospital attention.’ (Participant F, male, 35 years)

‘I once came on Saturday and they told me it’s not the time for treatment collection. Treatment is collected only during weekdays.’ (Participant G, male, 56 years)

**Sub-category 1.1.4.4: Poor infrastructure leading to a lack of privacy:** The participants stated serious concerns regarding the lack of privacy and confidentiality in the clinic setting.

They stated that they were attended to in shared spaces, where multiple patients are present simultaneously.

This exposes individuals’ health conditions and creates an environment in which sensitive information gets disclosed publicly. One of the participants said:

‘So, in our clinic, there is no privacy; we can be 3 in one room. Amongst these 3, I was the one who was infected. The nurse shouted at me, telling me that I’m going to infect the baby.’ (Participant A, female, 25 years)

Another participant put it in this way:

‘She came to me at the ward and asked me why I no longer took my treatment. She was so loud, and she screamed at me, saying, “Do you know that you are going to die if you don’t take your treatment! I felt that there was no privacy at all.”’ (Participant D, female, 27 years)

### Theme 2.1: Suggestions related to the improvement of adherence to anti-retroviral treatment

Participants suggested ways to improve ART adherence and reduce defaulters in Dr KK district. Suggestions related to improving adherence to ART emerged as the second theme, with only one category addressing improvements to the health system.

#### Category 2.1.1: Improvement of the health system

The category focused on improving health systems and consisted of four subcategories: treatment delivered to patients’ residential addresses, access to ART on weekends, nurses to demonstrate good attitudes, improvement of infrastructure and implementing a fast-lane model. Subthemes are discussed independently.

**Subcategory 2.1.1.1: Treatment delivered to patients’ residential addresses:** Some participants suggested that ART home delivery could help reduce defaulting, as many are unable to visit the facility. This is what one of the participants said:

‘If ever possible for people like us who are unable to get enough money to come to the clinic, we should have our treatment delivered to our home addresses.’ (Participant D, female, 27 years)

‘We should be able to collect our treatment on weekends because during the week we are at work and we are unable to collect treatment … weekend if they can operate the clinic just like during the week and again the community health worker should prepack our treatment and bring them for us in our respective residences.’ (Participant K, male, 38 years)

**Subcategory 2.1.1.2: Access to ART on weekends:** It has been discovered that the majority of patients seem to be working and they are unable to collect ART refills during the week, on the other hand, the facility does not fully allow patients to collect treatment. The participants reported a need to access ART on the weekends this is reported by the participants as follows:

‘On Saturday we could not get the treatment, now I have a piece job so when I come late, they say I must wait for 19h00 shift, when I knock off at 15h00 I do my vitals then again, I need to wait for 19h00 shift of nurses on the other hand I am hungry.’ (Participant G, male, 56 years)

Another one said:

‘We should be able to collect our treatment on weekends because during the week we are at work and we are unable to collect treatment, weekend if they can operate the clinic just like during the week.’ (Participant K, male, 38 years)

Other participants also suggested:

‘Department can make a provision for nurses to be on standby it can still be an in depended institute such as [*NGO*]. They can work from Monday to Friday until 16h00, then on weekend they can assist from 09h00 until 14h30 this will make a huge difference. Normally, on weekends when I leave the house around 08h00, I walk for about 20 minutes I will be able to get assistance as long as they have stipulated that they close at 14h30.’ (Participant F, male, 35 years)

**Subcategory 2.1.1.3: Nurses to demonstrate good attitudes:** The participants highlighted the critical role of healthcare workers’ attitudes and communication styles in shaping experiences of people living with HIV care and their adherence to ART. Negative behaviours such as shouting, speaking disrespectfully and disclosing patients’ HIV status in public spaces contribute to feelings of judgement, dehumanisation and stigma. Some of the participants’ suggestions are presented here, and they said:

‘Yes, they should be retrained, if possible, on how to treat patients. We are also human beings. I feel like someone is judging me when they assist me in a negative attitude. One other thing, nurses wherever they are, please encourage us to take our treatment.’ (Participant F, male, 35 years)

‘So, if they just need to learn to speak to us politely rather than shout at us and disclose our status in the public, I think in that way it will be better.’ (Participant A, female, 25 years)

‘According to my point of view, the manager of the clinic must call a meeting and talk to them about how to treat patients, not to talk to us inappropriately.’ (Participant B, female, 18 years)

**Sub-category 2.1.1.4: Improvement of infrastructure and implementing the fast-lane model:** The findings revealed that long wait times and the need to undergo routine procedures, such as vital sign assessments, are viewed as unnecessary and burdensome, even when patients are clinically stable and attending solely to collect their medications.

One of the participants said:

‘There is no need to be in a queue when you are done with vitals. You can just take your treatment and leave. I wish they could do it again, when it’s your date for collection, you would go straight to the window, there was no queue. You could just give them your card and sign. The treatment already has your name and your address. I think they should do like before where we used to receive an SMS to come and collect the treatment, that was meant for 3 months. From there I don’t know what happened.’ (Participant G, male, 56 years)

Another one put it this way:

‘If they could have a room for people who just collect treatment. When I come for collection, I just look for my name on the list then get my treatment. If we are too many in the line, we fight. I think we should go straight to the consulting room and not queue to take vitals because we are not sick. If you are sick, then you’ll have to stand in the queue but if you just came to collect treatment, you don’t see the need to queue.’ (Participant C, female, 40 years)

Another one said:

‘My last request the government should get us at least one consulting room that will be allocated to us so that we can be able to get our treatment quicker.’ (Participant H, male, 37 years)

## Discussion

One participant reported that she was unable to eat the whole day because of vomiting and dizziness, which suggests severe side effects of ART. Similarly, Nantambi and Suubi (2023:9) state that 64 (53.3%) participants defaulted on treatment because of ART side effects, including nausea, skin rashes and hallucinations. Ilesanmi and Afolabi (2021:141) concur that adherence may be impacted by toxicities and unfavourable side effects of ART regimens, ranging from mild to severe and from acute to chronic. The findings of the current study suggest that the lack of intervention on the signs and symptoms related to the severe side effects of ART exerts a negative impact on the general health of people living with HIV. Additionally, failure to treat the side effects and further referral of patients culminates in defaulting on ART.

In this study, socioeconomic barriers are highlighted as major contributors to ART default. The participants emphasise the challenge of transport costs, noting that long distances to the clinic, inadequate financial resources and relying solely on a social grant complicate and often compromise adherence to treatment. This reflects how structural poverty directly obstructs access to care, particularly in settings where healthcare facilities are geographically distant and transport is expensive. The participants described attending clinic appointments on an empty stomach as they could not afford even a small snack. Long waiting times exacerbate this vulnerability, resulting in fatigue, reduced concentration and difficulty engaging with nurses during consultations. Similarly, Twekambe et al. (2023:14) reported that patients defaulted on ART because of a lack of transport fares and food. Another study in Limpopo mentions that participants struggled to collect ART because of a lack of transportation funds and long distance from the medical institution (Lowane & Lebese 2022:4). These findings indicate that low socio-economic status is a significant barrier to accessing ART, as it contributes to food insecurity and discourages patients from returning for treatment because of a lack of money for transport.

This study found that community safety and environmental insecurity are significant barriers to consistent clinic attendance and ART adherence. Both participants describe living far from the clinic and facing serious safety risks when travelling, particularly during early morning or evening hours. While distance alone is noted, the primary concern is the threat of violence, including mugging, stabbings, sexual assault and gun violence, which creates fear and discourages travel to and from healthcare facilities. Contrary to the findings of this study, Chinuoya et al. (2014:114) state that people living with HIV and AIDS in South Africa have reportedly been subjected to street thugs who steal their Antiretroviral [ARV] and use them to make illicit drugs. Additionally, there have been reports of several primary health care facilities being broken into, with ARVs stolen, compromising the safety of patients attending clinics for ART refills (Chinuoya et al. 2014:114). The findings indicate that unsafe living environments pose a significant barrier to ART adherence, as patients feared travelling during late-night or early-morning hours to collect their treatment.

This study highlights work-related constraints as significant barriers to consistent ART collection and adherence. The participants described employment conditions characterised by irregular, complex and extended working hours, which limited their ability to attend clinic appointments scheduled during standard operating hours. Some employment involved abnormal shift patterns that conflicted with clinic visits, despite attempts to communicate these challenges to clinic staff. Becker et al. (2020:12) report that several participants explained that their supervisors and employers would not allow them to take time off, resulting in occasional missed clinic appointments or forgotten ART refills. In addition, some of the women also experienced threats of job termination by their superiors when they requested time off for a clinic appointment. The findings of the current study demonstrate that employers play a significant role in shaping employees’ adherence to chronic medications, including ART. Work environments characterised by inflexible schedules, long working hours and limited understanding of employees’ health needs certainly restrict opportunities to attend clinic appointments and collect medication, thereby increasing the risk of treatment default.

This study confirms intimate partner influence and relationship dynamics as significant factors contributing to ART default. The findings demonstrate how partners’ negative attitudes towards ART and HIV exert pressure that undermines adherence, particularly among women who may prioritise relationship stability over personal health. The decision to stop taking treatment was directly linked to a partner’s rejection of a female partner who uses ART. The same phenomenon was reported by Kasalu, Lazaro and Chilinda (2023:8), indicating that patients on ART without support from a loved one have an 11.25% increased risk of defaulting on ART than those with necessary support.

Shabalala et al. (2018:57) reported that ART might be difficult for some people to get, especially for women, because of fear of violence and/or rejection. The findings indicate that a lack of support from life partners is a significant interpersonal factor contributing to defaulting on ART. The findings further highlight the importance of emphasising partner involvement during ongoing counselling of people living with HIV to improve adherence.

The findings highlight excessive waiting times at the clinic as a major barrier to healthcare access.

The participants described spending an entire day at the facility, despite arriving early and, in some cases, living nearby. This suggests significant inefficiencies in service delivery, in which arrival time does not affect the speed or quality of care. Similarly, it has been observed that several patients default ART because of long waiting times at facilities (Becker et al. 2020:13). The patients who were dissatisfied with long queues were more likely to default their ART refill as they had to wait for 4–6 h (Becker et al. 2020:13). Although participants in the current study did not meet the eligibility criteria for registration in the CCMDD programme, the persistent overcrowding at the facility raises concerns about whether other patients on chronic treatment may also be ineligible. This underscores the need for regular screening and identification of patients eligible for enrolment in the CCMDD programme.

The findings indicate that negative attitudes and poor interpersonal relationships between nurses and people living with HIV play a significant role in influencing ART default. Most participants identified disrespectful behaviour from nursing staff as an additional factor contributing to their decision to discontinue ART follow-up. This was further confirmed by Becker et al. (2020:23), who showed that participants were humiliated when they arrived late at the facility, missing their appointment date, and the facility staff would never accept their plausible reasons for missing the ART collection date. The reported experiences of being shouted at in this study signify a lack of patient-centred and supportive care within ART services. These suggest that negative attitudes patently discourage patients from returning to health facilities, thereby increasing the risk of treatment interruption, poor viral suppression and adverse health outcomes.

The findings indicate that limited ART refill services on weekends create a structural barrier to access to treatment. Participants reported difficulty collecting ART because the facility prioritises emergencies on weekends and does not offer routine ART refills on weekends. As a result, patients may miss scheduled refills, increasing the risk of treatment interruption and non-adherence.

Similarly, Ilesanmi and Afolabi (2021:141) found that the inability to collect ART on weekends discourages patients from collecting their treatment, which may lead them to default, particularly if they are working all week. The authors further assert that it would be beneficial if clinics increased the capacity of health care workers so that ART clinics could accept working patients to collect ART on weekends in addition to weekdays.

The findings suggest that inadequate health facility infrastructure indirectly undermines adherence to ART by eroding patient privacy and confidentiality. Being attended to in shared consulting rooms exposes patients’ health information to others, which heightens fears of unintended disclosure and stigma. The same phenomenon was reported in a study conducted in Ethiopia, arguing that confidentiality is essential to successful ART as it increases adherence to treatment in people living with HIV (Bayisa et al. 2022:6). High ART defaulters attribute this to confidentiality breaches, with healthcare providers often disclosing client status without considering potential stigmatisation (Bayisa et al. 2022:6). These findings highlight the need for healthcare facilities to improve infrastructure by providing adequate private consulting spaces to protect patient confidentiality. Strengthening confidentiality measures certainly enhances patient trust, promotes open communication, improves adherence to ART and reduces treatment default rates.

In this study, participants suggested that treatment should be delivered to their households to reduce the rate of defaulters, as it tends to be difficult to collect it during the week. Similarly, another study demonstrated the sustainability and effectiveness of a community-based approach that involves delivering ART at home, and, on that note, community health workers receive training on confidentiality, consent, counselling and safe ART distribution (Hoke et al. 2021:2).

This study further attests that home delivery has been linked to reducing the default rates among people living with HIV, improving clinical outcomes, client satisfaction and cost savings for patients. This study suggests that home delivery of ART could be an effective strategy for eliminating barriers to clinical access.

Participants suggested that during ART visits, they desire to be treated with dignity and respect by the nursing staff. Ritshidze (2021:22) proposes that training must be conducted, and a circular issued requesting all employees to treat patients with dignity and to withdraw the policy of requiring people living with HIV who miss appointments to be sent to the back of the queue, as they need to be prioritised.

This study highlights the critical importance of respectful and dignified treatment in promoting adherence to ART among people living with HIV. When patients are treated with dignity and respect by nursing staff, they are more likely to feel valued, supported and comfortable in accessing healthcare services. This positive interaction fosters trust in healthcare providers and encourages continued engagement in care, which is essential for maintaining adherence to ART and achieving optimal health outcomes.

During interviews, the participants indicated that a separate consulting room would fast-track their visits. They further suggested that stable patients should not be placed in the queue, which will benefit patients during ART follow-up. In contrast to the findings of this study, Hoke et al. (2021:2) reported that people living with HIV should be integrated with general patients to help reduce stigma. The findings suggest that patients on ART should have access to dedicated consulting rooms, while service provision should be mindful of the risk of stigmatisation. The study also emphasises the need for a fast-track system for patients who are stable on ART.

### Limitations

Reaching the desired number of participants was a challenge, as the topic of the study was very sensitive; recruitment was difficult, as some patients within reach declined to take part.

During the recruitment process, some contact details in patients’ files were listed as voicemail, and during in-person tracing, most patients were not at home. These factors prolonged the data collection period. Some patients self-transferred to other clinics without following proper referral channels. Other patients who appeared on the list from Tier.Net were not defaulters; they were active on their carry books. However, there was missing information in the files, and treatment follow-up visits were not up to date in the clinical stationery. Data were collected in one facility in the North West province; therefore, results cannot be generalised to a broader population. For this reason, research must be conducted in other healthcare facilities.

### Recommendations

These are based on practice, nursing education, policy making and research.

Initially, the evidence that severe ART side effects contribute to treatment default highlights the need for strengthened clinical management of adverse drug reactions. Nursing staff must prioritise early identification, prompt treatment and appropriate referral of patients experiencing severe side effects.

Failure to adequately manage ART-related toxicities not only compromises patients’ physical well-being but also increases the likelihood of defaulting on ART by people living with HIV. This emphasises the importance of routine pharmacovigilance, patient education on expected side effects and flexible regimen adjustments to support sustained adherence.

The socio-economic barriers identified point to the need for broader social and structural interventions. Transport costs, long distances to clinics, food insecurity and reliance on social grants significantly restrict people living with HIV’s ability to access ART consistently. These findings imply that ART programmes must be integrated with social support mechanisms, such as transport assistance, food supplementation or decentralised service delivery models.

This study further highlights community safety and environmental insecurity as overlooked but critical barriers to ART adherence. Fear of violence while travelling to and from clinics discourages patients from attending appointments, particularly during early mornings or late evenings. This implies that service delivery models must prioritise patient safety, including flexible appointment times, closer-to-home ART services and community-based delivery options that minimise exposure to unsafe environments.

Work-related constraints identified in the findings suggest that rigid clinic operating hours and inflexible employment conditions significantly undermine adherence. This has implications for workplace and health system collaboration. Employers should be sensitised to the importance of supporting employees with chronic conditions, while health facilities should explore extended hours, weekend services or alternative ART refill options to accommodate working patients. Without such flexibility, employed patients remain at high risk of treatment interruption.

The influence of intimate partner relationships on ART adherence highlights the importance of addressing interpersonal dynamics in HIV care. The findings imply that ART programmes should strengthen counselling services that include partner involvement, where appropriate, and empower patients, particularly women, to prioritise their health without fear of rejection or violence.

Excessive waiting times and overcrowding at clinics point to systemic inefficiencies that discourage continued engagement in care. The findings imply a need for improved patient flow management, regular assessment of eligibility for differentiated service delivery models and expansion of programmes such as fast-track services for stable patients.

Negative attitudes and poor interpersonal relationships between nurses and people living with HIV have serious implications for treatment outcomes. These findings imply an urgent need for ongoing training in patient-centred care, supportive supervision and accountability mechanisms to promote respectful, non-judgemental interactions within ART services.

The limited availability of ART refill services on weekends represents a structural barrier that disproportionately affects working patients. Health facilities should reconsider service schedules and staffing models to ensure continuity of ART access beyond standard weekday hours. Expanding weekend services could reduce missed refills and improve adherence among employed patients.

Inadequate infrastructure and lack of privacy within health facilities have significant implications for confidentiality and stigma. These findings imply that investments in health facility infrastructure, particularly private consultation spaces, are essential for protecting confidentiality and promoting sustained ART.

### Nursing education

In higher education institutions, the undergraduate nursing curriculum should incorporate nurse-initiated management of anti-retroviral treatment (NIMART) training to expand the pool of nurses qualified to initiate and manage ART. Early exposure to NIMART during training may foster interest, build competence and enhance nurses’ ability to manage ART patients effectively, thereby supporting improved treatment adherence and patient outcomes.

### Research

Based on the findings of this study, further research could explore nurses’ understanding of addressing the emotional and psychological needs of patients on ART. In addition, studies focusing on developing strategies to strengthen support systems and improve access to ART for people living with HIV in the North West province are recommended. A quantitative study examining factors influencing treatment default among patients initiated on ART at the district level in the North West province is also necessary to allow for generalisation of findings to a larger population.

### Policy

Workplace HIV and AIDS policies should be strengthened to ensure that employers adequately accommodate employees with HIV-related illnesses by providing flexible leave arrangements.

## Conclusion

Based on the findings of this study, it is evident that people living with HIV face multiple factors that contribute to non-adherence to ART. These factors include severe ART side effects, low socioeconomic factors, unsafe living environments, negative attitudes from nurses, lack of support from life partners and inadequate infrastructure that compromises confidentiality. In this context, there is an urgent need to strengthen the early identification and management of severe ART side effects.

Additionally, the DoH should invest in improving the infrastructure of primary healthcare facilities to enhance privacy and protect the confidentiality of people living with HIV receiving ART.
